# Wnt/β-catenin signaling pathway: an attractive potential therapeutic target in osteosarcoma

**DOI:** 10.3389/fonc.2024.1456959

**Published:** 2025-02-14

**Authors:** Yi Ding, Qin Chen

**Affiliations:** ^1^ Department of Spine Surgery, Ganzhou People's Hospital, Ganzhou, China; ^2^ Department of Spine Surgery, Ganzhou Hospital-Nanfang Hospital, Southern Medical University, Ganzhou, China

**Keywords:** osteosarcoma (OS), Wnt/β-catenin, targeted therapy, chemotherapy, mechanism

## Abstract

Osteosarcoma (OS) is the most common bone malignancy in children and adolescents, and although current neoadjuvant chemotherapy has shown efficacy against OS, the long-term survival rate for patients with OS remains low, highlighting the need to find more effective treatments. In cancer cells, abnormal activation of signaling pathways can widely affect cell activity from growth and proliferation to apoptosis, invasion and metastasis. Wnt/β-catenin is a complex and unique signaling pathway that is considered to be one of the most important carcinogenic pathways in human cancer. Research have confirmed that the Wnt/β-catenin signaling pathway is an important driving factor for the occurrence and development of osteosarcoma, and abnormal activation of this pathway can promote the pathological processes of cell proliferation, invasion, migration, tumor angiogenesis and chemical resistance of osteosarcoma. However, inhibition of Wnt/β-catenin signaling pathway can effectively inhibit or reverse the above pathological processes. Therefore, manipulating the expression or function of the Wnt/β-catenin pathway may be a potential targeted pathway for the treatment of OS. In this review, we describe the characteristics of the Wnt/β-catenin signaling pathway and summarize the role and mechanism of this pathway in OS. This paper discusses the therapeutic significance of inhibiting or targeting Wnt/β-catenin pathway in OS and the shortcomings of current studies on this pathway in OS and the problems to be solved. This review helps us to understand the role of Wnt/β-catenin on OS, and provides a theoretical basis and new ideas for targeting Wnt/β-catenin pathway as a therapeutic target for OS.

## 1 Introduction

Osteosarcoma (OS) is the most common aggressive malignant bone tumor in children and adolescents worldwide (1). It originates from the original transformed cells of mesenchymal origin and mainly affects the differentiation of osteoblasts and produces immature bone (2, 3). Although the exact cause of OS is not fully understood, it is clear that the development and pathogenesis of OS is associated with multiple factors, including age, sex, ethnicity, genetics, and familial factors (4). OS is characterized by locally aggressive growth and high metastatic potential (5, 6), characterized by local invasion of bone and soft tissue, loss of function of the affected limbs, and distant metastasis, most often to the lungs (7). At present, the main treatments for OS are surgery, radiotherapy, neoadjuvant chemotherapy and postoperative adjuvant chemotherapy and other multi-scientific and multi-mode treatments, but the therapeutic effect is not satisfactory. Due to the early onset of bone and lung metastasis of OS, the 5-year overall survival rate of OS patients with metastasis at diagnosis is less than 30% (8, 9). More importantly, due to the complexity of the progression mechanism of osteosarcoma, the etiology and molecular mechanism of the pathogenesis are still vague or unknown. Therefore, there is an urgent need to further understand the physiological and pathological mechanism of OS, develop more effective anti-OS agents and new therapeutic strategies to improve the symptoms of OS from the molecular pathological level, so as to improve the prognosis and quality of life of osteosarcoma patients.

Although the cause of OS has not been fully elucidated, there is a large amount of evidence that the disease is related to the dysregulation of various intracellular signaling pathways, especially the Wnt/β-catenin pathway (10). Wnt/β-catenin signaling pathway has been reported to be one of the most important carcinogenic pathways in almost all human cancers (11) and seems to be a good candidate for molecular therapies in malignant tumors (12, 13). The activation of Wnt/β-catenin signaling pathway is also associated with the occurrence, development and pathological mechanism of a variety of diseases, such as cancer, Alzheimer’s disease, schizophrenia, diabetes and Parkinson’s disease (14–17). It has been found that overactivation of Wnt/β-catenin signaling pathway plays a carcinogenic role in various sarcomas by driving cell cycle progression and increasing cell proliferation (18–20). It has been confirmed that Wnt/β-catenin pathway is over-activated in osteosarcoma (21), and abnormal activation of this pathway can promote pathological processes such as cell cycle, migration, invasion, angiogenesis, chemotherapy resistance and accelerate the occurrence and development of OS, while inhibition of Wnt/β-catenin pathway activity can effectively inhibit the above processes (22). Therefore, targeting Wnt/β-catenin could provide a new perspective for designing more effective drugs for the treatment of OS. In this review, we reviewed the biological characteristics of Wnt/β-catenin, the role and mechanism of Wnt/β-catenin in the pathological process of OS. This paper aims to provide evidence for the effect of Wnt/β-catenin signaling pathway activation on OS and the treatment of OS, and to emphasize the role of inhibiting Wnt/β-catenin pathway in delaying OS by clarifying the mechanism, so as to provide theoretical basis and new ideas for targeting Wnt/β-catenin pathway as therapeutic targets for OS.

## 2 Summary of Wnt/β-catenin signaling pathway

Wnt/β-catenin signaling is an evolutionarily conserved signal that regulates many important embryonic and somatic processes such as cell fate determination, organogenesis, tissue homeostasis, and various pathological states (23, 24). In addition, the abnormal regulation of this signal transduction is often closely related to many aspects of tumor occurrence and development, malignant transformation and recurrence (23, 25–27). Wnt signaling can be classified into classical or non-classical pathways, the classical pathway is involved in cell survival, proliferation, differentiation and migration, while the non-classical pathway regulates cell polarity and migration (28). However, most current studies on the Wnt pathway focus on the Wnt/β-catenin branch of the Wnt pathway, the disorder of which is associated with a variety of diseases (29).

### 2.1 Classic Wnt pathway

**Figure 1:**
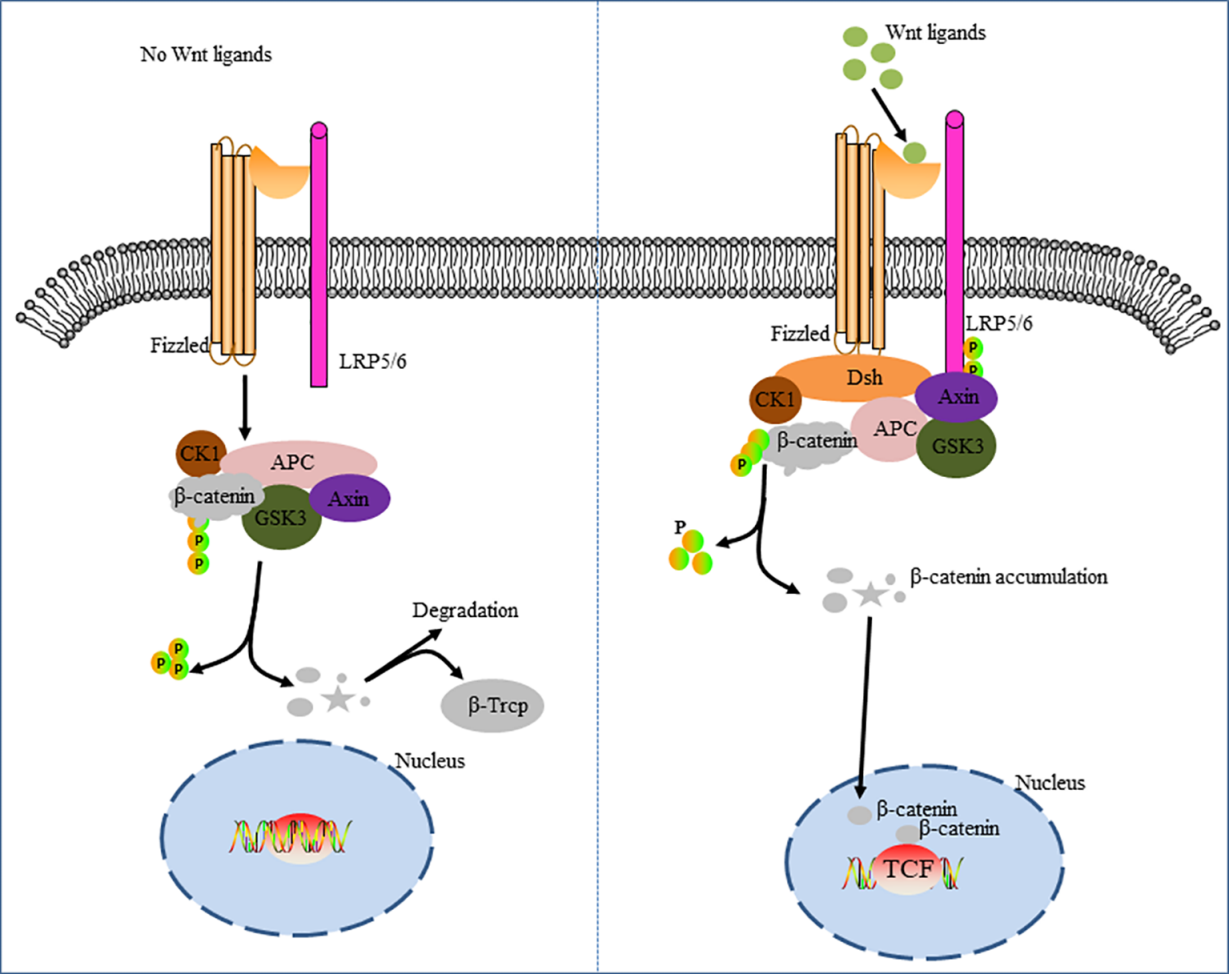
Classic Wnt/β-catenin pathway cascade diagram. The figure on the left shows the deactivation of the Wnt pathway. In the absence of Wnt signaling, β-catenin in the cytoplasm is recognized, folded, and phosphorylated by a destructive complex composed of scaffold proteins Axin, APC, GSK3β, and CK1, and targeted for degradation by a β-TRCP-mediated proteomic mechanism. The figure on the right shows activation of the Wnt pathway. The signal induces double phosphorylation of LRP6 by CK1 and GSK3-β via the Fizzled receptor and LRP5/6 co-receptor complex, which allows the axon-containing protein complex to transfer from the cytoplasm to the plasma membrane. Dsh is also recruited to the cell membrane and binds to Fizzied, and Axin binds to phosphorylated LRP5/6. The complex forms on the Fizzled/LRP5/6 membrane and induces stabilization of β-catenin by separating and/or degrading axons. β-catenin, which accumulates in the cytoplasm, translocates into the nucleus and, together with the transcription factor TCF, drives the expression of downstream target genes.

Classic Wnt pathway is a signal cascade mediated by β-catenin. The classical Wnt pathway is marked by the accumulation and translocation of the adherence-linked protein β-catenin in the nucleus. β-catenin is an Integral protein in the Wnt signaling pathway that regulates gene transcription and intercellular adhesion. Mutations in beta-catenin lead to amino acid substitution, resulting in inappropriate phosphorylation of the protein. Subsequently, ubiquitin ligase E3 does not correctly recognize the phosphorylated protein. Thus, dysregulation of the Wnt pathway leads to β-catenin accumulation without degradation and then transfer to the nucleus, thus activating transcription of oncogenes (30). Wnt is a secretory glycoprotein that binds to the cell surface transmembrane frizzled serpentine receptors (FZD) and low-density lipoprotein receptor-associated protein 5/6 (LRP5/6) complex, resulting in the accumulation of β-catenin in the nucleus, which leads to cell cycle activation and transcriptional regulation (31). FZD is the main binding site of Wnt (32), which mainly contains 7 transmembrane and extracellular N-terminal cysteine-rich domains (CRDS) (33, 34). However, in the absence of Wnt signaling, cytoplasmic β-catenin is degraded by β-catenin-destroying complexes such as Axin, adenomatous polyposis (APC), protein phosphatase 2A (PP2A), glycogen synthase kinase 3 (GSK3), and casein kinase 1α (CK1α). This results in the failure of the classical Wnt pathway (35, 36) (**Figure 1**).

### 2.2 Non-classical Wnt pathways

**Figure 2:**
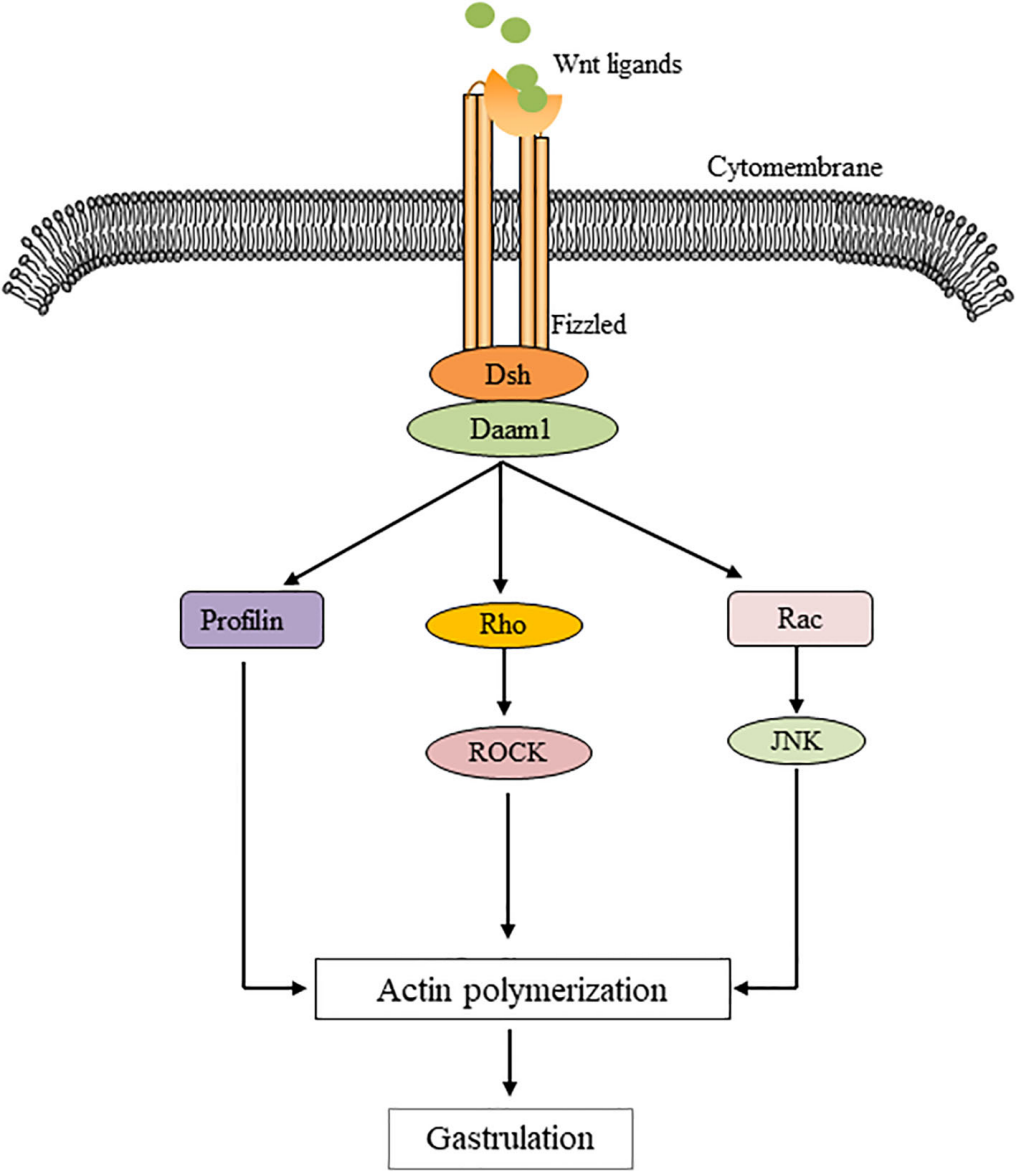
Illustration of a planar cell polarity transition cascade. Wnt signal transduction via Fizzled independent of LRP5/6 leads to Dsh activation. Dsh mediates the activation of Rho via Daam1, which activates Rho kinase (ROCK). Daam1 also mediates actin polymerization via the actin binding protein Profilin. Dsh also mediates the activation of Rac, which in turn activates JNK. Signals from Rock, JNK, and Profilin are integrated into cytoskeletal changes in cell polarization and movement during gastrum formation.

**Figure 3:**
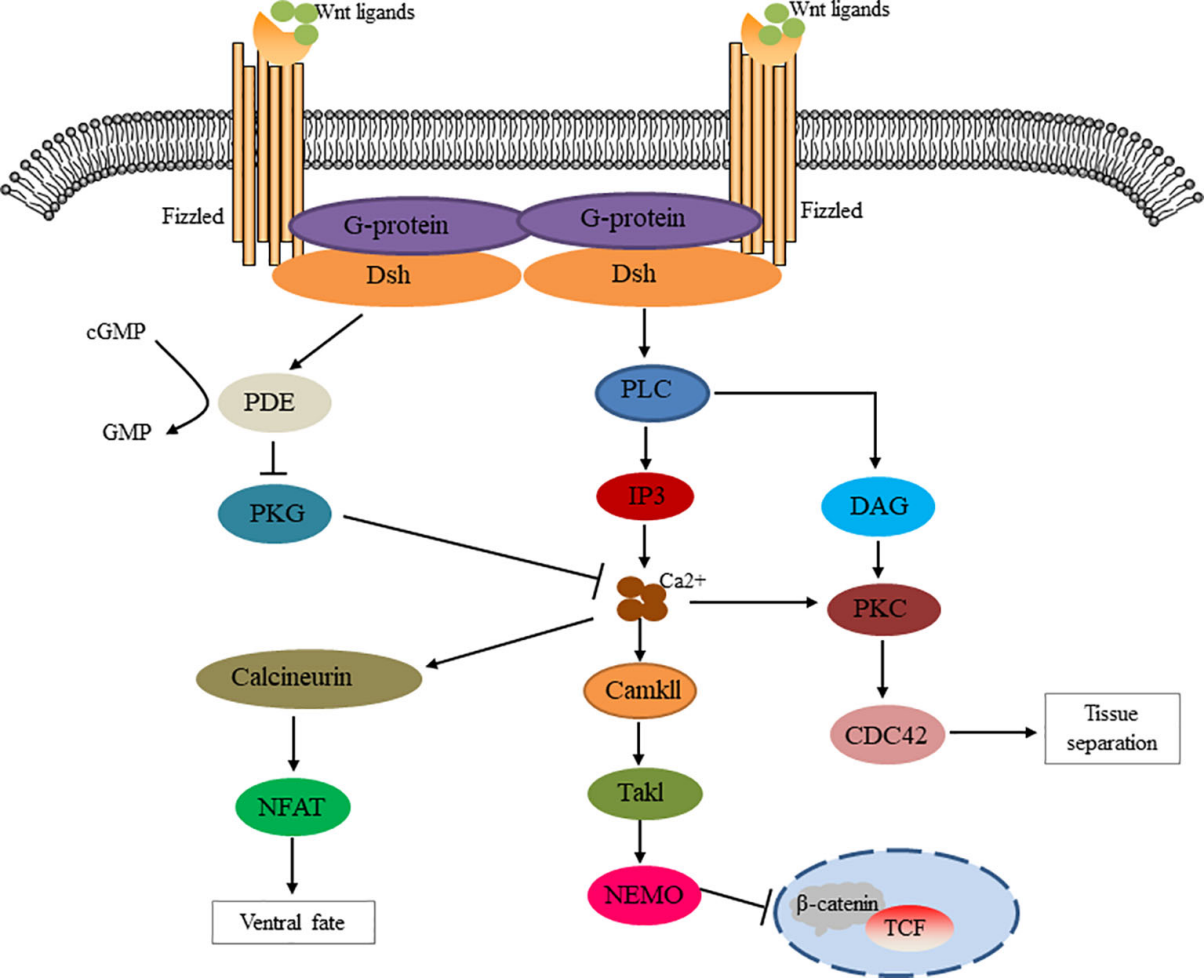
Schematic diagram of Wnt/Ca^2+^ signaling cascade. The Wnt signaling via Fizzled mediates the activation of Dsh through g protein activation. Dishevelled activates phosphodiesterase PDE, which inhibits PKG and in turn inhibits Ca^2+^ release. Dsh activates IP3 via PLC, resulting in the release of intracellular Ca^2+^, which activates CamK11 and calcineurin. NF-AT activation by calcineurin regulates the fate of ventral cells. CamK11 activates TAK and NLK, inhibits β-catenin/TCF function and negatively regulates dorsal axis formation. DAG mediates tissue separation and cell motility during gastrulation through PKC activation of CDC42.

The non-classical pathway is often referred to as the beta-catenin-independent pathway, which can be further divided into the Wnt/Planar Cell polarity (PCP) pathway and the Wnt/Ca^2+^ pathway. In the Wnt/PCP pathway, after the Wnt molecule binds to the receptor FZD, it recruits Dvl and further activates the small GTPases Rho and Rac, triggering the recruitment of downstream RHO-associated kinases (ROCK) and c-Jun N-terminal kinase (JNK), thereby allowing cytoskeletal recombination (**Figure 2**). In the Wnt/Ca^2+^ pathway, Dvl is activated when Wnt binds to FZD, and the activated Dvl recruits phospholipase C (PLC). PLC converts phosphatidylinositol 4, 5-diphosphate (PIP2) to diacylglycerol (DAG) and inositol 1,4, 5-triphosphate (IP3). IP3 stimulates the release of Ca^2+^ from the endoplasmic reticulum, and DAG and Ca^2+^ together activate downstream protein kinase C (PKC), calcineurin (CaN), and Ca^2+^/calmodulin-dependent protein kinase II (CaMKII), thereby regulating intracellular calcium flux and downstream calcium-dependent cytoskeleton and/or transcriptional responses (**Figure 3**).

## 3 Expression of Wnt/β-catenin signaling pathway in OS

Research have confirmed that the Wnt/β-catenin signaling pathway is involved in the development of osteosarcoma, and the Wnt signaling pathway is abnormally activated in OS and plays a crucial role in tumorigenic and metastatic transmission (37, 38). Wnt-β-catenin pathway has been reported to be significantly higher in human OS tissues and cell lines than in normal tissues or osteoid osteomas (39, 40). In addition, the analysis of relevant patient samples found that Wnt/β-catenin level in osteosarcoma tissues was significantly higher than that in neighboring healthy tissues, and was associated with poor prognosis and lung metastasis and diffusion (41, 42). Haydon et al. found that 33 out of 47 OS samples had increased Wnt/β-catenin expression level accumulation (43).

## 4 Role of Wnt/beta - catenin pathway in OS

**Figure 4:**
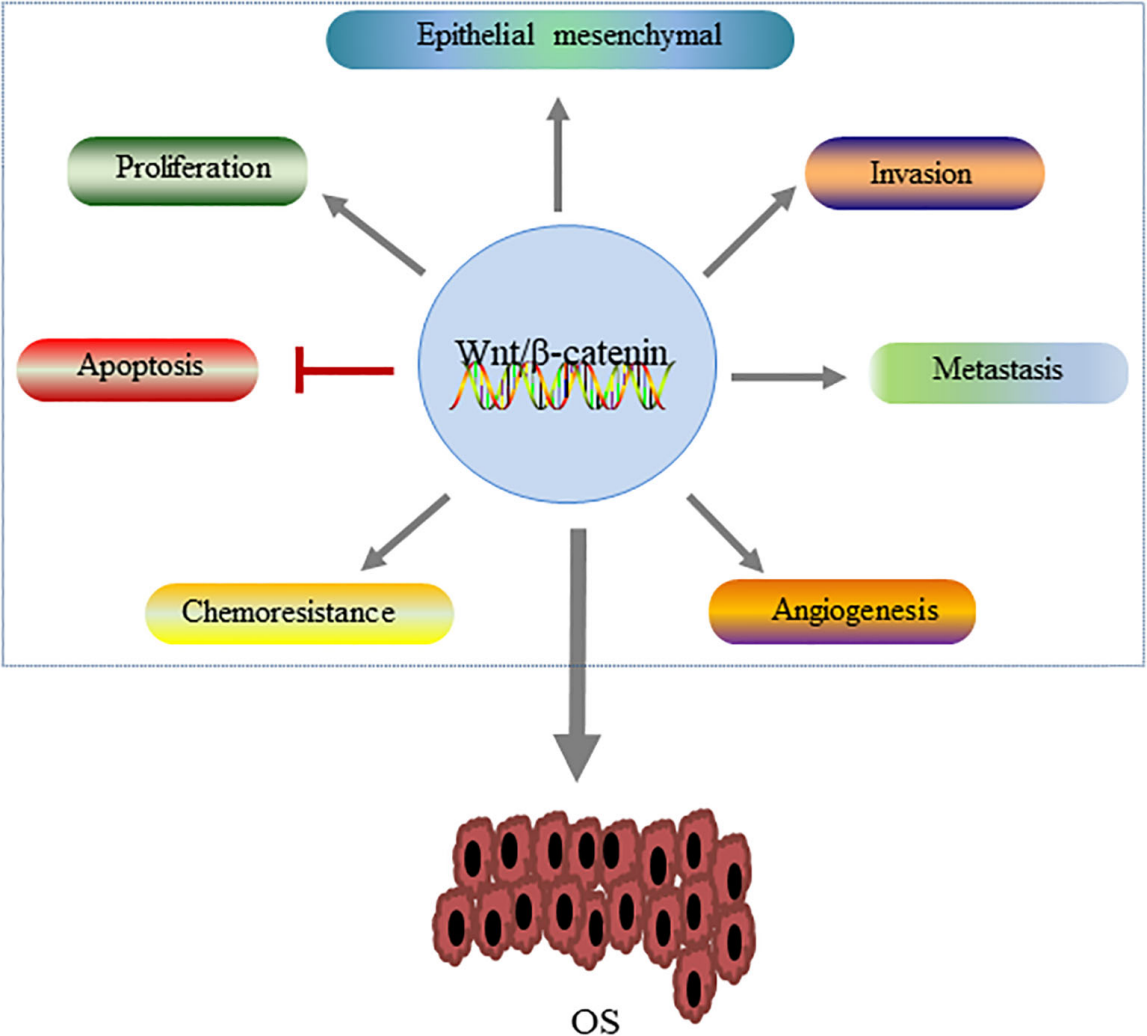
The role of the Wnt/β-catenin pathway in the development of OS. Activation of this signaling pathway regulates OS by promoting tumor cell proliferation, epithelial-mesenchymal transformation and tumor cell invasion and metastasis, angiogenesis and chemical resistance, and inhibiting apoptosis.

As an intracellular signaling pathway, Wnt/β-catenin pathway has been found in many types of cancer and plays an important regulatory role in the occurrence and development of tumors. Kinase analysis has identified active Wnt/β-catenin signaling in most OS cell lines (44). Wnt/β-catenin pathway is a complex signaling pathway, and its abnormal activation plays a key role in the pathogenesis of OS (45). A large amount of evidence has shown that dysregulation of this pathway is involved in multiple pathological processes of OS, including tumor cell proliferation, invasion and metastasis, and chemical resistance (46, 47) (**Figure 4**).

### 4.1 Promote the proliferation of tumor cells

The autonomous growth of cancer cells is usually controlled by changes in the expression of growth factors or growth factor receptors, resulting in cell proliferation. Many studies have shown that abnormal activation of Wnt/β-catenin signaling pathway plays a crucial role in the occurrence and development of osteosarcoma (38, 48, 49). Cell proliferation is an important feature of tumorigenesis and development, and also an important factor leading to poor prognosis. A large number of studies have shown that abnormal activation of Wnt signaling and high expression of β-catenin in OS are associated with abnormal histological morphology and cell proliferation and differentiation of osteosarcoma, ultimately leading to the occurrence of osteosarcoma (45, 50). When the level of β-catenin in the cytoplasm reaches a certain concentration, it begins to transfer to the nucleus and bind specifically to the nuclear transcription factor TCF/LEF, resulting in the exposure of downstream target gene promoter, activation and expression of promoter, causing abnormal cell proliferation and anti-apoptosis, thus promoting the formation of tumors (51). Huang et al. found that the Wnt/β-catenin pathway was activated in tumor tissues of patients with OS, and the activated Wnt/β-catenin pathway induced cell cycle progression and promoted the proliferation of OS cells. While cinnamaldehyde can inhibit Wnt/β-catenin signaling activity by down-regulating the phosphorylation of β-catenin and GSK-β as well as downstream target proteins c-Myc and MMP7, thereby inhibiting cell proliferation and promoting tumor cell apoptosis (52). In another study, activation of the Wnt/β-catenin pathway accelerated the progression of osteosarcoma, while inhibition of the activity of this pathway significantly inhibited the proliferation, migration, and invasion of osteosarcoma cells (53). In addition, Chen et al. (37) demonstrated abnormal activation of Wnt/β-catenin signaling in osteosarcoma cells, involving the autocrine Wnt signaling cycle and the upregulation of specific Wnt ligands and receptors. Activation of Wnt/β-catenin signaling with Wnt3a or GSK-3β inhibitors drives proliferation of osteosarcoma cells, whereas downregulation of activated Wnt signaling with dnTCF4 or siLEF1 inhibits proliferation and induces cell cycle arrest. From these studies, it was determined that activation of the Wnt/β-catenin pathway can promote cell proliferation in OS, thereby accelerating OS progression. Inhibition of this pathway can be an effective target to inhibit the progression of OS.

### 4.2 Inhibit apoptosis

Apoptosis is a kind of programmed cell death, which is an important process of normal development and tissue homeostasis. In many cancers, including osteosarcoma, apoptosis is the primary mechanism by which chemotherapy and radiotherapy induce cell death. The balance between cell proliferation and apoptosis in cancer cells is disturbed and leads to excessive proliferation of cancer cells through different molecular mechanisms (e.g. resistance to apoptosis). A large number of studies have shown that Wnt/β-catenin signaling pathway plays an important role in regulating the apoptosis process of tumor cells and is a key gene mediator involved in tumor cell apoptosis (12). Anti-apoptosis is a common feature of cancer cells, which is associated with increased expression of anti-apoptotic factors such as Bcl-2 or Bcl-xL or decreased expression, inactivation or mutation of pro-apoptotic factors such as Foxo3a or p53 (54). Interestingly, Wnt/β-catenin directly regulates an effective anti-apoptotic pathway and can also activate the expression of several anti-apoptotic genes, Bcl-2, Bad and Bcl-xl (55). Activation of the Wnt/β-catenin signaling pathway during cancer progression can promote the viability of osteosarcoma cells and inhibit apoptosis, and inhibition of this signaling can reverse this phenomenon (56). The mechanism may be through inhibiting Bcl-2 and promoting the expression levels of Bad and Bax (57). Studies have shown that miR-1-3p can deactivate Wnt/β-catenin signaling activity, thereby inhibiting the proliferation and cell cycle progression of osteosarcoma cells and promoting cell apoptosis (58). In addition, studies have also found that melittin is an anti-tumor Chinese medicine with few side effects, which can inhibit the activity of Wnt/β-catenin signaling pathway, up-regulate the ratio of Bax/Bcl-2 in osteosarcoma cells and inhibit the expression of proliferative protein, thus inducing apoptosis and inhibiting proliferation of tumor cells (59). These studies indicate that the abnormal expression of Wnt/β-catenin pathway plays an important role in the regulation of OS, and the activation of NF-κB pathway can inhibit the apoptosis of cancer cells. This allows targeting the Wnt/β-catenin pathway to eliminate the inhibition of OS cell apoptosis, so targeting Wnt/β-catenin may be a potential means to treat OS.

### 4.3 Promoting epithelial mesenchymal transformation and tumor cell invasion and metastasis

Epithelial mesenchymal transformation (EMT) is a process of migration of adherent epithelial cells to mesenchymal cells under certain conditions. It gives epithelial cells the characteristics of mesenchymal cells. In malignant tumors, EMT is closely related to the invasion and metastasis of tumor cells (60, 61). Proliferation and invasion play a crucial role in the progression of malignant tumors, including OS (7). There is growing evidence that the Wnt/β-catenin pathway promotes these aggressive behaviors (62, 63). On the other hand, osteosarcoma is an interstitial tumor, and patients with high malignancy tend to have distant metastases at an early stage and have a poor prognosis, in which EMT plays an important role (64). EMT is an important mechanism of embryogenesis, wound healing, fibrosis and other physiological processes (65–67), as well as an important process of distant metastasis and migration of tumor cells (68). Studies have shown that EMT plays an important role in the metastasis and migration progression of various tumor cells, including OS (69–71). It is well known that there are multiple interactions between molecular pathways and EMT mechanisms that promote cancer cell invasion. Many studies have shown that Wnt signaling pathway increases the invasion and malignancy of tumor cells through EMT induction (72–74). Wnt-induced EMT can enhance the proliferation of cancer cells and trigger their resistance to apoptosis (75, 76). The Wnt/β-catenin pathway is activated in OS, and the activated Wnt/β-catenin pathway promotes the migration and invasion of OS cells by enhancing the epithelial-mesenchymal transformation of OS (77). At the same time, activation of the Wnt/β-catenin pathway was associated with prognosis in patients with OS, and this study found that patients with over-activation of this pathway had significantly lower prognosis than patients with expression (78). Similar findings have been reported in another study of microRNAs on cancer occurrence and malignant progression in various tumors. This study found that inhibition of Wnt/β-catenin pathway activity could not only inhibit the EMT of OS, but also inhibit the progression and prognosis of OS (79). The invasion and metastasis of tumor cells are the main causes of death in patients with malignant tumors, and about 90% of patients with malignant tumors die from tumor metastasis (80). Studies on the prognosis of OS have found that the Wnt-β-catenin pathway can be used as a biological marker of metastasis (5). Lung metastasis is the most common site of osteosarcoma and the most common cause of death (81). Therefore, metastasis prediction is of great significance in the design of treatment strategies. Since the expression of β-catenin in the cytoplasm is significantly associated with the incidence of distant metastasis (82), β-catenin has been used as a biomarker for the potential of OS metastasis to the lung (81). β-catenin is a key component of the Wnt/β-catenin signaling pathway, relocating from the cytoplasm to the nucleus after Wnt ligand stimulation, thereby regulating gene expression (83). Wnt/β-catenin may also promote cancer cell migration and invasion by inducing the expression of transfer-related proteins such as intercellular adhesion molecule-1 (ICAM-1) and matrix metalloproteinases (MMPs) (84–88). It has been reported that inhibiting the Wnt/β-catenin pathway in OS cells MG-63 can inhibit the expression of MMP14, thus inhibiting the invasion and movement of MG-63 cells (89). In addition, Fu et al. (90) also found that the activation of Wnt/β-catenin in OS can promote the activity of MMP-9, and the enhancement of the activity of the latter can promote the invasion and metastasis of OS cells. In addition, KLF5 is a positive regulator of the Wnt/β-catenin signaling pathway, and KLF5 can increase β-catenin expression and interact directly with β-catenin to stabilize it and promote its nuclear transfer (91). ML264 is a small molecule inhibitor of KLF5, and ML264 can inhibit the activity of Wnt/β-catenin signaling pathway, thereby inducing G0/G1 cell cycle arrest and inhibiting the migration and invasion ability of osteosarcoma cells (92). Based on the above studies, it can be determined that the activation of Wnt/β-catenin can promote the invasion and migration of OS, and inhibiting the activation of Wnt/β-catenin signaling pathway may be a potential therapeutic target to prevent the deterioration of osteosarcoma.

### 4.4 Promote angiogenesis

Angiogenesis is a complex biological process that leads to the development of new blood vessels and plays a key role in the progression and metastasis potential of various malignant tumors (93). In the process of angiogenesis, VEGF is the most important factor of angiogenesis, which can increase capillary permeability and promote the migration of tip cells (94). MMPs accelerated the proliferation and differentiation of endothelial cells cultured on type IV and type I collagen in a dose-dependent manner (95). It has been confirmed that Wnt/β-catenin signaling pathway plays an important role in the regulation of tumor angiogenesis. Studies have shown that activation of Wnt/β-catenin pathway can promote cytoskeletal recombination and new blood vessel formation of endothelial cells, possibly through promoting the expression of VEGF and MMPs (96, 97). In addition, transcriptional regulation of VEGF by β-catenin/TCF complex involves TCF binding sites in VEGF gene promoters. Activation of Wnt/β-catenin can induce VEGF overexpression, thus promoting angiogenesis (98, 99). However, there are relatively few studies on the role of Wnt/β-catenin pathway in the angiogenesis of OS, and its mechanism has not been fully cleared. However, it can be preliminarily confirmed that the abnormal expression of NF-κB pathway can promote the angiogenesis of OS.

### 4.5 Chemoresistance

Neoadjuvant therapy and adjuvant chemotherapy in addition to radical surgery have been shown to significantly improve the prognosis of patients with osteosarcoma. At present, the treatment of OS patients is mainly based on radical surgical treatment with standard three-drug chemotherapy regimen (including doxorubicin, cisplatin and high-dose methotrexate) to improve the survival rate (100). However, chemotherapy can create chemotherapy resistance and even lead to disease recurrence or metastasis (101). There are several mechanisms of drug resistance in chemotherapy, including reduced intracellular drug accumulation, drug inactivation, increased DNA repair, and signal transduction pathway perturbation (102). Some studies have found that abnormal activation of the Wnt-β-catenin signaling pathway is involved in chemotherapy resistance of OS cells, especially resistance to standard three-drug chemotherapy. Doxorubicin is an anthracycline antibiotic that is widely used to treat a variety of cancers, including OS. Wnt-β-catenin signaling targeting T-cell factor represses syndecan-2, a key modulator of apoptosis and chemosensitivity in OS cells, contributing to the resistance of OS to doxorubicin (103, 104). Methotrexate (MTX) is another common component of chemotherapy regimens for osteosarcoma. MTX resistance is a problem in OS chemotherapy and one of the mechanisms underlying MTX resistance is associated with Wnt/β-catenin signaling. Ma and colleagues (105) found that knocking down β-catenin increased the sensitivity of Saos2 cells to MTX-induced cell death. Thus, Wnt/β-catenin signaling may contribute to MTX resistance. In addition, it has been found that activation of the Wnt/β-catenin pathway in human OS cells can induce cell resistance to cisplatin, and the use of Wnt/β-catenin pathway inhibitors can improve or reverse the resistance of OS cells to cisplatin (106, 107). In summary, the Wnt/β-catenin pathway is often abnormally expressed in OS and regulates the pathophysiological processes of osteosarcoma cell proliferation and apoptosis, invasion, migration, tumor angiogenesis, and chemical drug resistance.

## 5 The effect of crosstalk between Wnt/β-catenin and other signal pathways on OS

In addition to the abnormal expression of Wnt/β-catenin pathway, abnormal activation of other signaling pathways, such as phosphoinositol 3-kinase/protein kinase B (PI3K/AKT) and NF-κB signaling pathways, also play an important role in the pathogenesis of OS. Signaling pathways may interact, and enhancement of one signaling pathway may enhance or inhibit the other. In the process of tumor formation and development, Wnt/β-catenin signaling pathway can directly or indirectly interact with other signaling pathways to regulate the pathophysiological processes of OS. This section describes crosstalk between Wnt/β-catenin and the PI3K/Akt and NF-κB pathways in OS.

### 5.1 PI3K/Akt signaling pathway

Phosphatidylinositol 3-kinase/protein kinase B (PI3K/Akt) signaling pathway is an important signal transduction bridge connecting extracellular signals and cellular responses (108). Phosphatidylinositol 3-kinase (PI3K) is a large class of signaling lipases, a family of enzymes that phosphorylates the 3’-OH of phosphatidylinositol inositol rings (109). Protein kinase B (Akt), an evolutionarily conserved serine protein kinase of the serine/threonine kinase family, is a central mediator in PI3K signaling (110). The PI3K/Akt signaling pathway is a highly conserved signal transduction network in eukaryotic cells and can promote cell survival, cell growth and cell cycle progression (111, 112). It has been reported that the PI3K/Akt signaling pathway is a common activation pathway in human cancer, and it is believed that the dysfunction of this pathway will drive the occurrence and development of cancer and participate in the regulation of cancer pathological process (113–115).

The PI3K/Akt and Wnt/β-catenin signaling pathways are generally not abnormally activated in a variety of tumors, including osteosarcoma (116, 117). These two pathways play an important role in the occurrence and development of osteosarcoma by regulating cell cycle, inhibiting apoptosis, promoting angiogenesis, enhancing metastasis and inducing chemotherapy resistance (118, 119). GSK-3β is a key protein in the downstream PI3K/Akt pathway (120), and activation of PI3K/Akt pathway can significantly inhibit the expression of GSK-3β (121). GSK3-β phosphorylates serine/threonine residues in β-catenin proteins, thereby promoting ubiquitination and subsequent proteasome-mediated β-catenin degradation (41). In addition, it has been found that overexpression of Wnt5a in non-transforming Wnt family members can stimulate the migration of osteosarcoma MG63 cells by promoting the phosphorylation of PI3K and Akt (122). In addition, another transforming member of the Wnt family, Wnt7b, activates mTORC1 via PI3K-AKT signaling, thereby promoting bone formation (123). Therefore, we speculated that PI3K/Akt and Wnt/β-catenin signaling pathways in OS regulate each other, and jointly participate in the regulation of the pathological process of OS.

### 5.2 NF-κB signaling pathway

As an intracellular signaling pathway, NF-κB signaling pathway plays a key role in a variety of physiological and pathological processes, such as inducing immune and inflammatory responses and regulating apoptosis (124–126). The NF-κB signaling pathway has been reported to be an important carcinogenic pathway in human cancers. Dysregulation of this pathway has been found in many types of cancer, such as prostate cancer, colorectal cancer, bladder cancer, breast cancer, and osteosarcoma (127–131). It has been reported that the NF-κB pathway is over-activated in osteosarcoma, leading to excessive proliferation of tumor cells and accelerated development of OS (132). In addition, abnormal expression of this pathway is widely involved in cell processes such as proliferation, apoptosis, cycle, chemotherapy resistance, and metastasis of tumors, including osteosarcoma (133, 134).

Studies have shown that Wnt/β-catenin pathway components can regulate inflammation and immune responses through interaction with NF-κB (135). NF-κB has been shown to indirectly regulate Wnt/β-catenin by regulating target genes that affect β-catenin activity or stability (136). Several studies have shown that the NF-κB and Wnt signaling pathways collaborate at multiple levels in different physiological and pathological contexts. In breast cancer cells, beta-catenin binds to the p65-p50 complex and inhibits its nuclear translocation (137). β-TrCP1 simultaneously activates NF-κB and inhibits the Wnt pathway in vascular smooth muscle cells (138). IKKα and IKKβ are key activators of the NF-κB pathway, which regulate Wnt/β-catenin signaling activity in different ways (139). IKKα inhibitors block the expression of CCND1, a downstream Wnt gene, in mouse embryonic fibroblasts (136).

## 6 Targeted Wnt/β- catenin pathway treatment OS

At present, the main treatment strategies for OS are surgery, radiotherapy and chemotherapy. Although current neoadjuvant chemotherapy has shown efficacy against OS, the long-term survival rate for patients with OS remains low, highlighting the need to find new treatments. As an important pathway regulating cell growth, metabolism, survival, and chemotherapy resistance, targeting the Wnt/β-catenin pathway may be a potential therapeutic approach for patients with OS. This section focuses on the therapeutic effects of non-coding RNAs and drugs on OS by regulating the Wnt/β-catenin signaling pathway.

### 6.1 Non-coding RNA

Non-coding RNA plays an indispensable role in the growth and development of organisms through its influence on transcription and translation. Abnormal expression of NcRNAs has been shown to affect the development and evolution of OS disease. MiRNA s are highly conserved ncRNAs that can affect mRNA expression by binding to the 3′ untranslated region of mRNA (3′UTR) (140). It was found that multiple miRNAs were overexpressed in OS. For example, miR-21-5p (141) and miR-374a (142) are upregulated in OS cells and tissues and promote OS cell migration by activating Wnt/β-catenin signaling, while downregulation of these miRNAs can inhibit the activity of Wnt/β-catenin to inhibit the progression of osteosarcoma. In addition, some miRNAs are underexpressed in OS and participate in the regulation of the pathological process of OS by activating the Wnt/β-catenin signaling pathway. For example, decreased miR-22-3p and increased miR-22-3p in OS tissues and cells inhibit the Wnt/β-catenin pathway by targeting TCF7L2, thereby preventing the progression of osteosarcoma (143). In addition, the expression of miR-1-3p is decreased in osteosarcoma tissues and cells, and upregulation of miR-1-3p inhibits the proliferation and cell cycle process of osteosarcoma cells by targeting CDK14 and inactivating Wnt/β-catenin signaling, while promoting cell apoptosis (58). In addition to the above miRNAs, the overexpression of miR-199b-3p (144) and miR-377-3p (79) can also inhibit the progression of osteosarcoma by inhibiting the Wnt/β-catenin signaling pathway. In addition, overexpression of miR-140 may inhibit the proliferation of human OS cells and may enhance drug sensitivity by directly regulating Wnt/β-catenin signaling (145).

Circular RNAs (circRNAs) are a class of non-coding RNAs characterized by covalently closed loops that have been found in a variety of diseases, including cancer (146). It was found that the expression of various circRNAs in OS was up-regulated and involved in the regulation of its pathological process. For example, Circ_0003732 is up-regulated in osteosarcoma tissues and cells, and activates the Wnt/β-catenin signaling pathway by regulating the miR-377-3p/CPEB1 axis, promoting the proliferation, migration and invasion of osteosarcoma cells, and inhibiting apoptosis. Silencing circ_0003732 can reverse the effect on the progression of osteosarcoma cells (147). The expression of hsa_circ_0087302 is low in osteosarcoma cells, and overexpression of hsa_circ_0087302 can inhibit the proliferation, cell cycle, migration and invasion of osteosarcoma cells by inhibiting the Wnt/β-catenin signaling pathway (148). In addition, circUBAP2 expression is upregulated in osteosarcoma tissues and cells, and knock down circUBAP2 can act as a sponge of miR-506-3p to inhibit Wnt/β-catenin signaling pathway activity, thereby inhibiting cell proliferation, migration and invasion, and promoting apoptosis of cisplatin-resistant osteosarcoma cells (149).

Long non-coding RNAs (LncRNAs) are endogenous ncRNAs with a length > 200 nucleotide transcripts and do not have protein-coding capabilities (150). By acting as a miRNA molecular sponge and competitively binding to miRNA, it is involved in a variety of *in vivo* pathophysiological processes, including cancer proliferation and invasion (151, 152). UCA1 influences the invasion and migration of osteosarcoma cells by mediating the Wnt/β-catenin pathway through the miR-145/HMGA1 axis (153). lncRNA SNHG10 is overexpressed in OS and acts as a sponge of miR-182-5p to activate the Wnt/β-catenin signaling pathway, promoting the proliferation, migration and invasion of osteosarcoma cells, while downregulation of SNHG10 can inhibit the above results (154). MRPL23-AS1 competitively interacts with miR-30b to activate the Wnt/β-catenin pathway, thus promoting tumorigenesis and metastasis of OS (155). In addition, HOTAIR (156), LncRNA FLVCR1-AS1 (53), CASC15 (157), and LINC00665 (158) are all upregulated in OS cells and affect the cell cycle by activating the Wnt/β-catenin pathway, thereby promoting cell proliferation (**Table 1**).

**Table 1:** Non coding RNAs associated with Wnt/β-catenin signaling pathway in OS.

NcRNAs	Mechanisms	Reference
miRNAs		
miR-21-5p	miR-21-5p↓→ Inhibit Wnt/β-catenin pathway → Inhibit cell proliferation and migration	(141)
miR-374a	miR-374a↓ → Inhibit Wnt/β-catenin pathway → Inhibit cell proliferation and migration	(142)
miR-22-3p	miR-22-3p↑ → Target TCF7L2 → Inhibit Wnt/β-catenin pathway → Inhibit proliferation and cell cycle of osteosarcoma cells and promoted apoptosis	(143)
miR-1-3p	miR-1-3p↑ → Target CDK14→Inhibit Wnt/β-catenin pathway → Inhibit proliferation and cell cycle of osteosarcoma cells and promoted apoptosis	(58)
miR-199b-3p	miR-199b-3p↑ → Inhibit Wnt/β-catenin pathway → Inhibit proliferation and cell cycle of osteosarcoma cells and promoted apoptosis	(144)
miR-377-3p	miR-377-3p↑ → Inhibit Wnt/β-catenin pathway → Inhibit proliferation and cell cycle of osteosarcoma cells and promoted apoptosis	(79)
miR-140	miR-140↑ → Inhibit Wnt/β-catenin pathway → Enhanced drug sensitivity	(145)
circRNA		
Circ_0003732	Circ_0003732↓→Regulation of miR-377-3p/CPEB1 axis → Inhibit Wnt/β-catenin pathway → Inhibit the proliferation, migration and invasion of osteosarcoma cells, and promote cell apoptosis	(147)
Hsa_circ_0087302	hsa_circ_0087302↓ → Inhibit Wnt/β-catenin pathway → Inhibit the proliferation, cell cycle, migration and invasion of osteosarcoma cells	(148)
CircUBAP2	circUBAP2↓ → Sponges acting as miR-506-3p → inhibit Wnt/β-catenin pathway → Inhibit cell proliferation, migration and invasion, and promoted apoptosis of cisplatin-resistant osteosarcoma cells	(149)
lncRNA		
lncRNA UCA1	lncRNA UCA1↓ →inhibit Wnt/β-catenin pathway → Inhibits cell invasion and migration	(153)
lncRNA SNHG10	lncRNA SNHG10↓→Act as a sponge for miR-182-5p → inhibit Wnt/β-catenin pathway → Inhibit proliferation, migration and invasion of cancer cells	(154)
MRPL23-AS1	MRPL23-AS1↓ → MRPL23-AS1 competitively interacts with miR-30b→Inhibit Wnt/β-catenin pathway → Inhibit cell proliferation and migration	(155)
HOTAIR	HOTAIR↓ → inhibit Wnt/β-catenin pathway→ Inhibits cell cycle and cell proliferation	(156)
LncRNA FLVCR1-AS1	LncRNA FLVCR1-AS1↓→ Inhibit Wnt/β-catenin pathway→ Inhibits cell cycle and cell proliferation	(53)
CASC15	CASC15↓ → inhibit Wnt/β-catenin pathway → Inhibits cell cycle and cell proliferation	(157)
LINC00665	LINC00665↓ → inhibit Wnt/β-catenin pathway→ Inhibits cell cycle and cell proliferation	(158)

↓, Expression down; ↑, expression up.

### 6.2 Drugs

At present, it has been found that many drugs can participate in the regulation of the pathological process of OS by inhibiting the activity of Wnt/β-catenin signaling pathway. For example, Resveratrol is a natural phenol (159). It has been reported that the treatment of resveratrol can arrest the cell cycle of various malignant tumors, promote cell apoptosis and inhibit the proliferation of cancer cells (160). In addition, resveratrol inhibits cell growth and induces senescence in OS cells by altering DNA metabolism (161). Zou et al. (30) found that resveratrol could inhibit the expression of β-catenin and c-Myc protein and mRNA, thereby inhibiting the proliferation of OS cells. Another study also came to a similar conclusion that resveratrol can inhibit the activity of Wnt/β-catenin signaling pathway and down-regulate the levels of c-myc, cyclin D1, MMP-2 and MMP-9, thus promoting apoptosis and inhibiting the proliferation and invasion of OS cells (162). Curcumin is a natural compound that comes from the roots of turmeric. Its anti-cancer properties have been demonstrated in many types of cancer, including OS (163, 164). Curcumin has been reported to delay the progression of osteosarcoma by regulating the Wnt/β-catenin pathway in osteosarcoma cells (165). Leow et al. found that curcumin analogs could inhibit the activity of Wnt/β-catenin pathway and prevent the invasion of osteosarcoma cells (90). Baicalein, a flavonoid extracted from the root of scutellaria baicalensis, has been proven to play an anti-tumor role by inhibiting the metastasis of various cancers and inducing apoptosis (166, 167). Studies have shown that baicalin can delay the progression of osteosarcoma by inhibiting the Wnt/β-catenin signaling pathway (168). Melatonin is a natural derivative of tryptophan, an amino acid with various biological activities (169), which plays a key inhibitory role in the pathogenesis of various types of tumors (170, 171). Li et al. (172) found that melatonin inhibited the expression of lncRNA JPX by regulating the Wnt/β-catenin pathway, thereby inhibiting the progression of OS. In addition, Oridonin (173), dihydroartemisinin (174), polyfolin I (175) and oleandrin (176) can also inhibit cell proliferation and induce apoptosis by inhibiting the activity of Wnt/β-catenin pathway, thus inhibiting the progression of OS (**Table 2**). Based on the above study, it is not difficult to find that targeting the Wnt/β-catenin pathway may be an innovative approach to treat osteosarcoma.

**Table 2:** Drugs associated with Wnt/β-catenin signaling pathway in OS.

Drugs	Mechanisms	Reference
Resveratrol	Inhibit Wnt/β-catenin pathway → Inhibit the expression of c-Myc, Cyclin D1, MMP-2 and MMP-9 → Promote OS cell apoptosis, inhibit cell proliferation and invasion	(162)
Curcumin	Inhibit Wnt/β-catenin pathway → inhibit cell proliferation and invasion	(90)
Baicalein	Inhibit Wnt/β-catenin pathway → Inhibit metastasis and induce apoptosis	(168)
Melatonin	Inhibit Wnt/β-catenin pathway→Inhibit the expression of lncRNA JPX → Inhibit OS progression	(172)
Oridonin	Inhibit Wnt/β-catenin pathway→Inhibit cell proliferation and induce apoptosis	(173)
Dihydroartemisinin	Inhibit Wnt/β-catenin pathway→Inhibit cell proliferation and induce apoptosis	(174)
Polyfolin I	Inhibit Wnt/β-catenin pathway→Inhibit cell proliferation and induce apoptosis	(175)
Oleandrin	Inhibit Wnt/β-catenin pathway→Inhibit cell proliferation and induce apoptosis	(176)

### 6.3 Combination treatment regimen

Due to the complexity of the tumor microenvironment, it is difficult to control the progression and recurrence of cancer with a single traditional treatment. Therefore, the combination treatment strategy has gradually become an inevitable trend in cancer treatment (177). Through combination therapy, Wnt/β-catenin signaling pathway inhibitors combined with other drugs can promote therapeutic efficacy and improve prognosis. Hattinger CM et al. used lithium carbonate in combination with neoadjuvant chemotherapy and 9-ING-41 in combination with doxorubicin to effectively inhibit GSK3-β, thereby enhancing the inhibitory effect on Wnt/β-catenin signaling pathway activity (178). In addition, preclinical studies by Leow et al. showed that inhibiting the Wnt/β-catenin pathway with curcumin and PKF118-310 reduced nuclear β-catenin levels, which in turn reduced intrinsic and activated β-catenin/TCF transcriptional activity and, consequently, the expression of β-catenin target genes. This resulted in the down-regulation of MMP-9, a reduction in the expression of cyclin-D, c-MYC, and survivin, and inhibition of the potential for migration. This had a suppressive effect on cell proliferation and increased cell mortality (165).

## 7 Discussion and future outlook

Wnt/β-catenin signaling pathway is an intracellular signaling pathway that is finely regulated, and abnormal expression of this pathway plays a crucial role in the occurrence and development of a variety of malignant tumors, including OS. Structural activation of Wnt/β-catenin is a novel hallmark of various tumor types, and many *in vitro* and animal models have shown that this pathway is involved in multiple steps of cell proliferation, apoptosis, epithelial-mesenchymal transformation, tumor angiogenesis, invasion, and metastasis through complex molecular mechanisms. At present, under the multi-science and multi-mode therapy, the research work to improve the effect of chemotherapy has led to the improvement of the survival rate of patients, but the prognosis of OS is not satisfactory, so molecular targeted therapy has gradually attracted widespread attention. Combined with the role of the Wnt/β-catenin pathway in the progression of OS, it can be seen that this pathway may be a potential target for OS therapy, and targeting the Wnt/β-catenin pathway may be an innovative approach for the treatment of osteosarcoma and a potential target for cancer drug development.

### 7.1 Limitations of current research on the Wnt/β-catenin signaling pathway

Most existing preclinical trial reports suggest that the Wnt/β-catenin pathway plays an important role in OS. Since Wnt/β-catenin signal plays a variety of functions in the pathological process of OS, it is generally difficult to verify the specific role of this signaling pathway in OS. Current epidemiological studies on Wnt/β-catenin and OS have some limitations, including lack of depth of study design, incomplete study subjects, and traditional targeted drug delivery. At a macro level, most of the current research designs on the Wnt/β-catenin pathway in the field of OS are still at the stage of cell or rodent research, and different experimental conditions and modeling will inevitably produce different experimental results. At the same time, there is a lack of clinical research in this field. At the micro level, current studies have focused on the protein expression of the Wnt/β-catenin pathway, rather than the gene and single-cell level. The development of OS is a complex process involving multiple molecular signaling pathways, so the interaction of the Wnt/β-catenin signaling pathway with other pathways in OS is also noteworthy. In terms of drug delivery, although some drugs are already in the preclinical research stage, the route of administration largely determines the therapeutic effect, efficacy and safety of drugs (179). Due to the first-pass effect of oral administration, only a small amount of the active ingredient can reach the designated site. Multiple adverse reactions may also be induced, thus limiting clinical application (180).

### 7.2 Challenges in translating Wnt/β-catenin targeted therapies

Wnt/β-catenin is expressed not only in cancer cells, but also in healthy cells, which may lead to unexpected effects of treatment. Therefore, one of the major challenges facing the use of Wnt/beta-catenin inhibitors in the treatment of osteosarcoma is safety and efficacy. Blocking or disabling Wnt/β-catenin signaling may impair immunity or induce toxic reactions. In addition to the development of cancer, many other factors, such as microbial infections or physical and chemical damage, can cause damage to the body (181). Therefore, systemic administration of Wnt/β-catenin inhibitors may worsen immune function or response. The degree of toxicity depends on the dose, time, and baseline health of the patient. Strategies to mitigate these toxicities and improve safety therefore include dose optimization and the use of modified or alternative Wnt/β-catenin-targeting drugs (182). Tumor heterogeneity is also a challenge for Wnt/β-catenin targeted therapies, as some cancer cells may have different levels of Wnt/β-catenin expression, which can lead to differences in the sensitivity of different cells to Wnt/β-catenin targeted therapies. In addition, due to the presence of immunosuppressive cells such as tumor-associated macrophages and tumor-associated neutrophils, cancer cells can upregulate immune checkpoint molecules to evade immune surveillance and escape immune cell destruction, which interferes with the effectiveness of Wnt/β-catenin targeted therapy (183). At presently, some challenges remain existed in the treatment accurately targeting the Wnt/β-catenin signaling pathway. The biggest obstacle is devoid of reliable predictive biomarkers that can identify patients who will be most likely to benefit from these types of therapy.

To overcome these challenges, a variety of strategies are being developed. One approach is to combine targeted therapies with other immune checkpoint inhibitors to overcome the immune escape mechanism adopted by tumor cells (184). Another approach is to develop bispecic antibodies that can simultaneously target Wnt/β-catenin and another immune checkpoint molecule. Finally, other advanced and efficient techniques and methods should be used to enhance the efficacy of Wnt/β-catenin targeted therapy.

### 7.3 Current and future research trends of Wnt/β-catenin pathway in OS

The mechanism of action of the Wnt/β-catenin pathway in OS has been widely discussed, but there is still much to be improved. First, due to the lack of large-scale multi-center clinical trials, the specific molecular mechanisms leading to the activation or inhibition of the Wnt/β-catenin signaling pathway during OS have not been cleared. Therefore, future studies should focus on large-scale clinical studies and pharmacological studies to further explore the specific mechanism of action of Wnt/β-catenin signaling pathway in OS, and strive to provide reliable medical evidence for the development of clinical treatment for patients with OS. Second, current studies targeting the Wnt/β-catenin pathway for OS treatment have been too conventional. Future studies are needed to determine how to link targeted Wnt/β-catenin with corresponding front-line therapies for OS with improved drug delivery strategies leading to internalization to achieve *in vivo* drug delivery, targeted drug release, and biological activity to enhance drug administration efficiency, improve therapeutic effectiveness, and maximize targeting and reduce resistance. It is continuously released to enhance the efficiency of drug use, improve the therapeutic effect and reduce side effects (185). In particular, mesenchymal stem cells, nanoparticles and hydrogels are used. The main limitation of nanoparticles, however, is their toxic profile. Due to the wide variety of nanoparticles, the toxicity characteristics of nanoparticles are not well characterized, and the toxicity assessment of each class of these nanoparticles requires great efforts.

## 8 Conclusion

As an intracellular signaling pathway, abnormal activation of Wnt/β-catenin signaling pathway can promote the proliferation of osteosarcoma cells, epithelial-mesenchymal transformation, invasion and metastasis, tumor angiogenesis, and chemical resistance of tumor cells, and inhibit the activity of Wnt/β-catenin signaling pathway can delay the progression of osteosarcoma. Based on the role of the Wnt/β-catenin pathway in the development of OS, we suggest that targeting or manipulating the expression or function of the relevant Wnt/β-catenin signaling pathway may be an innovative approach to the treatment of OS and a potential target for cancer drug development.
